# Optical coherence tomography imaging of the basal ganglia: feasibility and brief review

**DOI:** 10.1590/1414-431X20154679

**Published:** 2015-09-29

**Authors:** W. O. Contreras Lopez, J. S. Ângelos, R. C. R. Martinez, C. K. Takimura, M. J. Teixeira, P. A. Lemos, E. T. Fonoff

**Affiliations:** 1 Divisão de Neurocirurgia Funcional, Departamento de Neurologia, Faculdade de Medicina, Universidade de São Paulo, São Paulo, SP, Brasil; 2 Laboratório de Neuromodulação e Dor Experimental, Hospital Sírio-Libanes, São Paulo, SP, Brasil; 3 Instituto do Coração, Universidade de São Paulo, São Paulo, SP, Brasil

**Keywords:** Optical coherence tomography (OCT), Basal ganglia, Rat brain, Brain imaging, Histology, Thalamus

## Abstract

Optical coherence tomography (OCT) is a promising medical imaging technique that uses light to capture real-time cross-sectional images from biological tissues in micrometer resolution. Commercially available optical coherence tomography systems are employed in diverse applications, including art conservation and diagnostic medicine, notably in cardiology and ophthalmology. Application of this technology in the brain may enable distinction between white matter and gray matter, and obtainment of detailed images from within the encephalon. We present, herein, the *in vivo* implementation of OCT imaging in the rat brain striatum. For this, two male 60-day-old rats (*Rattus norvegicus*, Albinus variation, Wistar) were stereotactically implanted with guide cannulas into the striatum to guide a 2.7-French diameter high-definition OCT imaging catheter (Dragonfly™, St. Jude Medical, USA). Obtained images were compared with corresponding histologically stained sections to collect imaging samples. A brief analysis of OCT technology and its current applications is also reported, as well as intra-cerebral OCT feasibility on brain mapping during neurosurgical procedures.

## Introduction

At present, deep brain stimulation (DBS) procedures require extreme precision because the therapeutic effects of focal electrical stimulation in neuropsychiatric diseases highly correlate with electrode placement in specific brain targets (1). Because of the need for localization and target verification, those procedures are commonly performed using computed tomography/magnetic resonance imaging (CT/MRI)-guided stereotactic methods after frame attachment to the skull. These image sets have relatively low resolution and are usually acquired before the procedure starts. For instance, CT/MRI updating during the procedure is not always possible and, if possible, tends to be extremely time-consuming.

Intra-operative microelectrode recordings are routinely performed to map extracellular neuronal activity according to brain area; the recordings also provide submillimetric resolution based on particular cell-firing patterns (2). Physiological mapping is quite expensive, highly demanding, time-consuming, and there is the risk of complication caused by microelectrode penetration (3). Additionally, stereotactic procedures and microelectrode recordings are performed with conventional two-dimensional atlases and require paper records of neurophysiological data (4). This approach is prone to errors, especially for subcortical structures.

In this context, optical coherence tomography (OCT) is a novel image technique that provides high-resolution images using infrared light (5) *in situ* and in real time (6), and appears to be an interesting additional tool for deep-brain navigation. Here, we present a brief review of OCT technology and its clinical applications. We also report preliminary results from imaging of the basal ganglia of *Rattus novergicus* using OCT.

## Material and Methods

**Figure 1:**
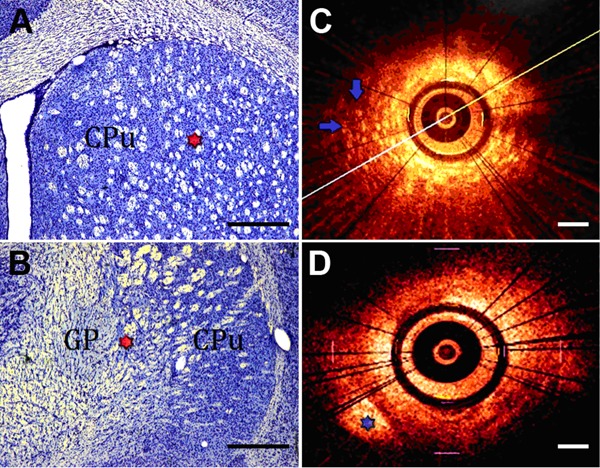
Histological and optical coherence tomography (OCT) images of CPu [caudate-putamen (striatum)] and GP (globus pallidus). *A*, *B*, coronal sections of rat brain striatum and their corresponding *in vivo* images on the right side (*C*, *D*). OCT images identified tissue types based on the interface between the gray basal nuclei and white matter. Red stars in *A* and *B* indicate chosen target; in *C,* the blue arrows point out a striosome pattern; in *D*, the blue star indicates the GP, which corresponds to the target on the red star (*B*). The scale bar corresponds to 200 μm.

Two male 60-day-old rats (*Rattus norvegicus*, Albinus variation, Wistar) were used in compliance with the recommendations of the Brazilian Society of Neuroscience and Behavior, which, in turn, are based on the US National Institutes of Health Guide for Care and Use of Laboratory Animals. Animals were anesthetized before surgery with an intraperitoneal injection of 0.09 mL xylazine + 0.1 mL morphine + 0.3 mL ketamine, and then placed in a stereotactic frame (David Kopf Instruments, Germany) with the incisor bar set at -3.3 mm below interaural zero. Caudate-putamen (CPU) and globus pallidus internus (GPi) stereotactic coordinates were determined with references to Bregma, according to the stereotactic atlas of Paxinos and Watson (7). Targets were: CPU (AP 0.6 mm, ML 3.5 mm, DV -5.0 mm) and GPi (AP -1.56 mm, ML 4.0 mm, DV -7.0 mm). A stainless metal cannula was adjusted to the stereotactic frame guiding a modified fiber optic OCT catheter (Dragonfly™, St. Jude Medical, USA) that was connected to a mobile console (C7-XRTM, St. Jude Medical) for OCT image acquisition. As mentioned above, structures with a diameter of 2-3 mm were scanned, and real-time video was recorded to illustrate OCT trajectory. Images were processed and then compared with rat brain histological slices of the corresponding studied nucleus available (Figure 1).

## Results and Discussion

OCT was able to identify tissue types in rat basal ganglia nuclei and delineate their positions in real time, as shown in Figure 1. It also could identify striatal cells that appear as irregular individual structures with different signal attenuation according to myelin content, thus enabling differentiation between white matter (high signal attenuation) and gray matter, which conversely appears as signal-rich protruding structures. Additionally, OCT provided the potential to determine the next type of tissue boundary.

In principle, OCT imaging is analogous to the ultrasound B-mode imaging, except that it uses light rather than acoustic waves. Cross-sectional images are generated by scanning an optical beam across the tissue and measuring the echo time delay and intensity of backscattered light (8). This is because OCT is an interferometric technique that measures scattering light from the tissue with a near-infrared wavelength optical beam (700-1000 nm) (9). Image resolutions of 1-15 μm can be achieved, which are 10-100 times greater than conventional ultrasound imaging, MRI, or CT. OCT imaging, however, is limited to 2-3 mm in depth by optical attenuation and scattering (8).

Beginning almost 25 years ago, OCT started its journey into the mainstream of ophthalmology, initially developed commercially for retinal and vitreo-retinal interface diseases and glaucoma (10), because of the transparent properties of the anterior eye and retina. To date, OCT has had the largest clinical impact in high-resolution retinal imaging (6).

Imaging gastrointestinal structures with OCT, for example, enables visualization of histological morphology in real time, especially the epithelial structures, such as villi, crypts, squamous, and intestinal epithelium (11). In cardiology (12), the *in vivo* visualization of vulnerable atherosclerotic plaques can now be enhanced with high-resolution OCT imaging, which is also able to detect the lumen diameter in relation to vulnerable plaques and stent characteristics.

In neurosurgery, OCT has been reported to discriminate between healthy and pathological human brain tissue (13), and has been used to image a cadaveric human cortex with a metastatic melanoma (5) and to test the suitability of OCT to guide stereotactic procedures in the brain (14). This last application, if validated, could dramatically improve electrode implantation for deep-brain stimulation (DBS), so that targeted nucleus would be visualized in real time.

Jafri et al. (14) determined that optical guidance for stereotactic procedures, such as DBS, relies on the ability to optically detect junctions of white matter presented in cerebral tracts and gray matter of deep brain nuclei to use them as landmarks for the target. Strong backscattering and poor penetration characterize the white matter, resulting in a bright appearance on the OCT images.

These peculiar fiber and nuclei aspects could function as important features during electrode descent in brain tissue, even for deep targets. The subthalamic nucleus, for example, could be easily assessed by an OCT probe, because its anatomical boundaries are defined by dense bundles of myelinated fibers (15). This could improve the benefits of focal electrical stimulation and avoid adverse clinical effects associated with electrode misplacement.

Using a different system, Jeon et al. (3) also conducted *in vitro* experiments in the rat brain using optical properties of OCT, selecting regions with white and gray matter junctions to be scanned and prepared for a histological view. Internal structures were visualized and matched to corresponding locations in the rat brain atlas, which enabled the identification of the external capsule, optic tract, internal capsule, hippocampus, and lateral geniculate nucleus. The authors concluded that OCT renders clear tissue classification among different tissue types, favoring the placement of deep-brain electrodes.

The small range of tissues that can be imaged by OCT, as a result of low light penetration, could signal the limitations for its use when a wider surgical view is necessary. However, these drawbacks can be overcome if the probe can be inserted close to the target structure, as suggested by recent advances in OCT technology that enable three-dimensional image reconstruction soon after acquisition (16).

This initial analysis aimed to correlate OCT images with a histological view of the striatum and its transition to the globus pallidus. This reinforces the window opened by OCT for the development of studies using this technology in the human brain.

OCT technology enabled the visualization of rat brain basal ganglia *in vivo* and in real-time, similar to histological views. These results suggested that this technique could be used as a precise and sensitive imaging method.

The feasibility provided by a simple system apparatus and the possibility to combine with current stereotactic tools may enable the use of this technique in the human brain, thereby improving patient safety and target precision. Human intracerebral use will require, however, a more detailed understanding of normal OCT neuroimaging. Therefore, further experimental studies are important and necessary for safety evaluation and real clinical impact.
